# Quantitative fit analysis of acromion fracture plating systems using three-dimensional reconstructed scapula fractures – A multi-observer study

**DOI:** 10.1051/sicotj/2021028

**Published:** 2021-05-20

**Authors:** Johan Charilaou, Roopam Dey, Marilize Burger, Sudesh Sivarasu, Ruan van Staden, Stephen Roche

**Affiliations:** 1 Department of Surgery, Division of Orthopaedic Surgery, Groote Schuur Hospital 7935 Cape Town South Africa; 2 Department of Human Biology, Division of Biomedical Engineering, University of Cape Town 7935 Cape Town South Africa; 3 Faculty of Medicine and Health Sciences, Division of Orthopaedic Surgery, Stellenbosch University 7935 Cape Town South Africa

**Keywords:** Acromion fracture, Scapula plates, Clavicle plates, Quantitative fit, Additive manufacturing

## Abstract

*Introduction*: Surgical treatment of displaced acromial and scapula spine fractures may be challenging due to the bony anatomy and variable fracture patterns. This difficulty is accentuated by the limitations of the available scapular plates for fracture fixation. This study compares the quantitative fitting of anatomic scapular plates and clavicle plates, using three-dimensional (3D) printed fractured scapulae. *Methods*: Fourteen scapulae with acromion and spine fractures were used for this study. Computerized tomographic (CT) scans of the fractured scapulae were obtained from the Philips picture archiving and communication system (PACS) database of patients admitted to a tertiary teaching hospital in Cape Town, South Africa between 2012 and 2016. The reconstructed scapulae were 3D printed and the anatomical acromion and clavicle plates were templated about the fracture regions. The fit assessment was performed by five observers who classified the plates as no-fit, intermediate fit, and anatomical fit according to the surgical guidelines. *Results*: The 6-hole anterior clavicle plate performed better than any of the scapular plates as they were able to fit 45.7% of the fractured acromion, including the spine. Among the pre-contoured anatomical scapula plates, both the short and the long acromion plates could fit only 27.3% of the fractured acromion. The intraclass correlation coefficient was 0.965 suggesting excellent consensus among the five observers. *Conclusion*: Clavicle plates were found to be better suited to fit around a scapula fracture in its acromion and spine region.

## Introduction

Acromion fractures are uncommon and only comprise 8–16% of all scapula fractures [1, 2]. Fractures of the scapula process are generally managed non-operatively with satisfactory to excellent outcomes [3]. Surgical outcomes, involving scapula fractures, are reported in abundance [4–7] but reports on alternative methods of fracture fixation using scapula plates are sparse and small case series [1]. Even though these studies suggest that scapular fractures, involving scapular spine and acromion, can be treated surgically [8–10] there are no large studies investigating the performance of the available commercial anatomical scapula reconstruction plates.

Separate studies performed on 12 [11] and 5 [1] scapulae reported satisfactory outcomes after open reduction and internal fixation (ORIF) of fractured scapulae using these scapula reconstruction plates. Anecdotal experience suggests that pre-contoured plates designed for a specific anatomical region can be implanted on different periarticular regions with similar morphometry. We have been using the lateral clavicle reconstruction plates for these fractures. Other surgeons have also reported their use of these plates for internal fixation of the acromion fractures [1, 12] when the plate-bone congruency is not optimal [11]. It is important to note that the acromion region has attachment points deltoids and important rotator cuff muscles such as supraspinatus pass underneath it, therefore if fractures of this process are not well fixed the function of the whole shoulder complex can be compromised [13]. There are no studies in the literature that extensively investigates the performance of these plates for fractures observed in Southern Africa. We have access to limited suppliers of anatomical scapula plates which put us at a disadvantage compared to the developed countries. The primary aim of this study was to perform a quantitative fit analysis of acromion and clavicle plates on scapulae with acromial and spine fractures. The secondary aim of this study was to compare the performance of the acromion and clavicle plates.

## Materials and methods

A multi-observer quantitative fit analysis of the acromial and clavicular plates on scapula fractures was performed at a tertiary hospital. Ethical and institutional approval from the relevant committees were obtained. Computerized tomographic (CT) scans of all patients with scapula fractures referred to the tertiary hospital between 2012 and 2016 were identified using the Philips picture archiving and communication system (PACS). The CT scans were examined and patients with acromion and spine fractures were identified. Patients with fractures due to gunshot wounds were excluded as the fracture patterns were comminuted and did not fit the usual fracture classifications.

Fourteen scapulae with acromion and spine fractures were included. The average age at injury for the selected patients was 42.3 years (range: 23–74 years). There was a male predominance (93%, *n* = 13) and commonly (60%, *n* = 9) right-sided injuries. Pedestrian vehicle accidents accounted for 46.7% (*n* = 7) of the injuries, followed by motor vehicle accidents (40%, *n* = 6), and lastly due to blunt trauma (13.3%, *n* = 2).

**Figure 1:**
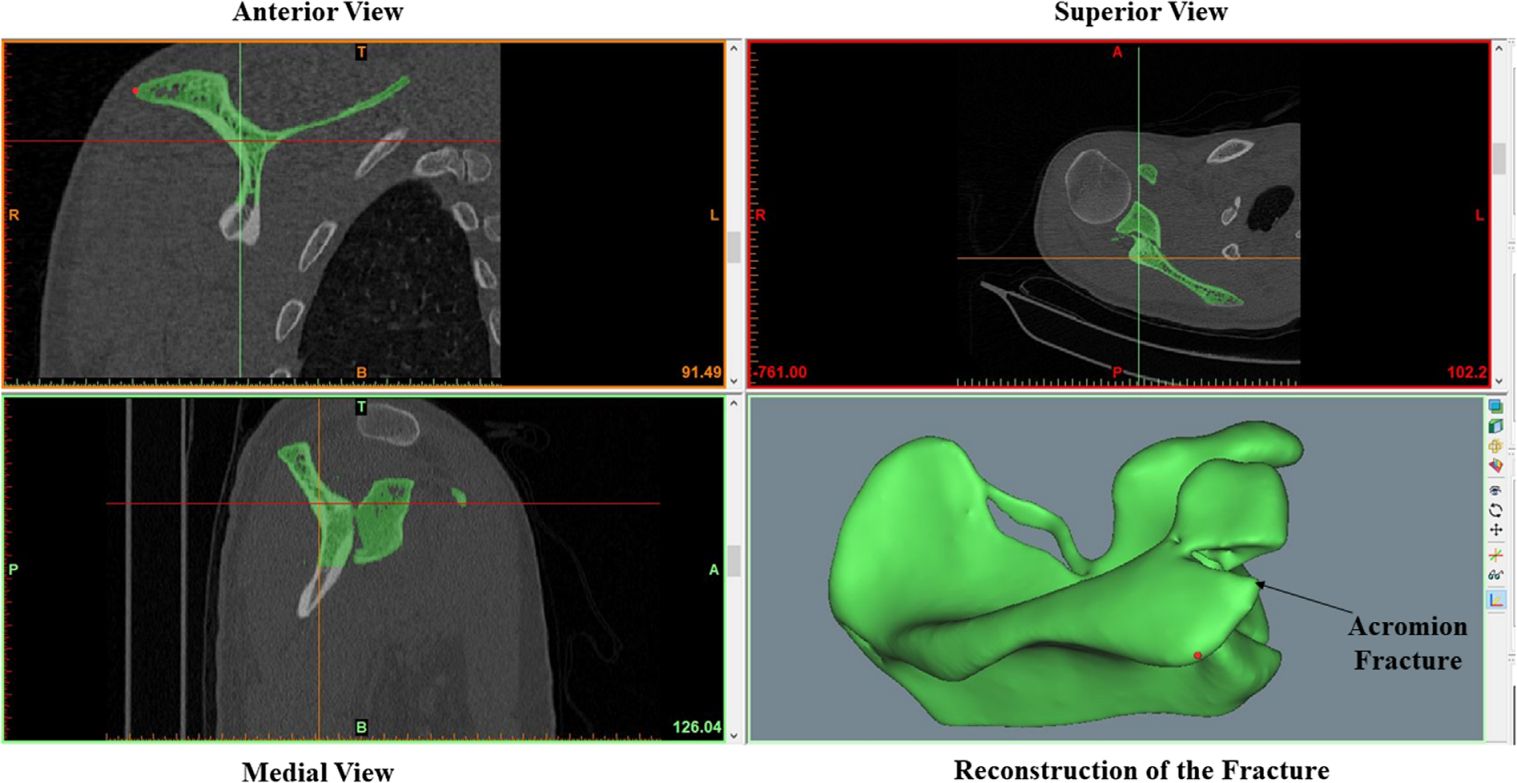
The bone reconstruction platform of Materialise Mimics^®^ that was used for all the fractured scapula in this study.

The Digital Imaging and Communication in Medicine (DICOM) files of the acromion fractures were obtained from the CT scans. These DICOM images were made to create individual *in-silico* three-dimensional (3D) models of the fractured scapulae using Materialise Mimics^®^ (Leuven, Belgium) (Figure 1). The individual fractured models of scapula were 3D printed using a commercial additive manufacturing device. Acrylonitrile Butadiene Styrene (ABS), a common thermoplastic polymer filament was used to construct the scapulae during the additive manufacturing process. The use of CT reconstruction and 3D printing has been previously employed by various other studies [14, 15].

**Figure 2:**
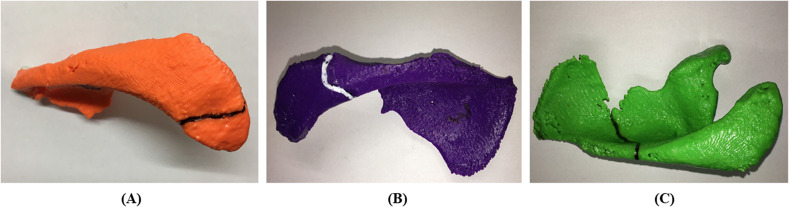
This image shows the type of fracture patterns that were encountered in this study. The fracture lines are highlighted using marker. (A) Type I fracture; (B) type II fracture; (C) type III fracture.

The fracture patterns were identified on each 3D printed model and were classified into three types according to the anatomical location of the fracture propagation (Table 1). The fracture fragment was anatomically reduced and kept in this position using adhesive with the fracture line traced with ink on the surface (Figure 2). This process was repeated for all 14 scapulae.

**Table 1:** Details about the acromion fracture types encountered in this study.

Type	Fracture site description	Frequency (%)
Type I	Acromion process lateral to acromion angle.	2 (14.3)
Type II	Acromion angle to spinoglenoid notch.	4 (28.6)
Type III	Medial to the spinoglenoid notch or into scapular spine.	8 (57.1)

**Figure 3:**
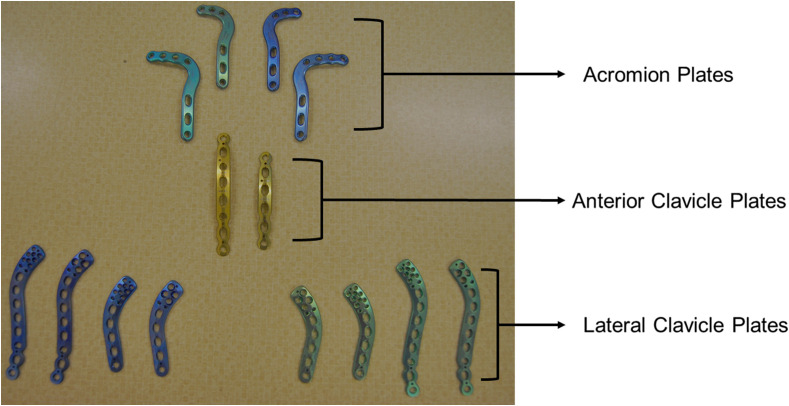
The complete set of plates used in this quantitative fit analysis of acromion fracture.

Eight plates, including two acromion and six clavicle plates, were used in this study (Figure 3). These plates were sourced from the only manufacturer (Acumed^®^) available in our country. Plates were templated on the fractured region of the acromion by five independent observers. These observers comprised of a specialist shoulder surgeon, a trainee orthopedic surgeon, a medical student, a biomedical engineer, and a biokinetist. They individually templated the plates on each scapula and rated the quantitative fit of each plate around the acromion fracture line on two occasions, two weeks apart.

**Figure 4:**
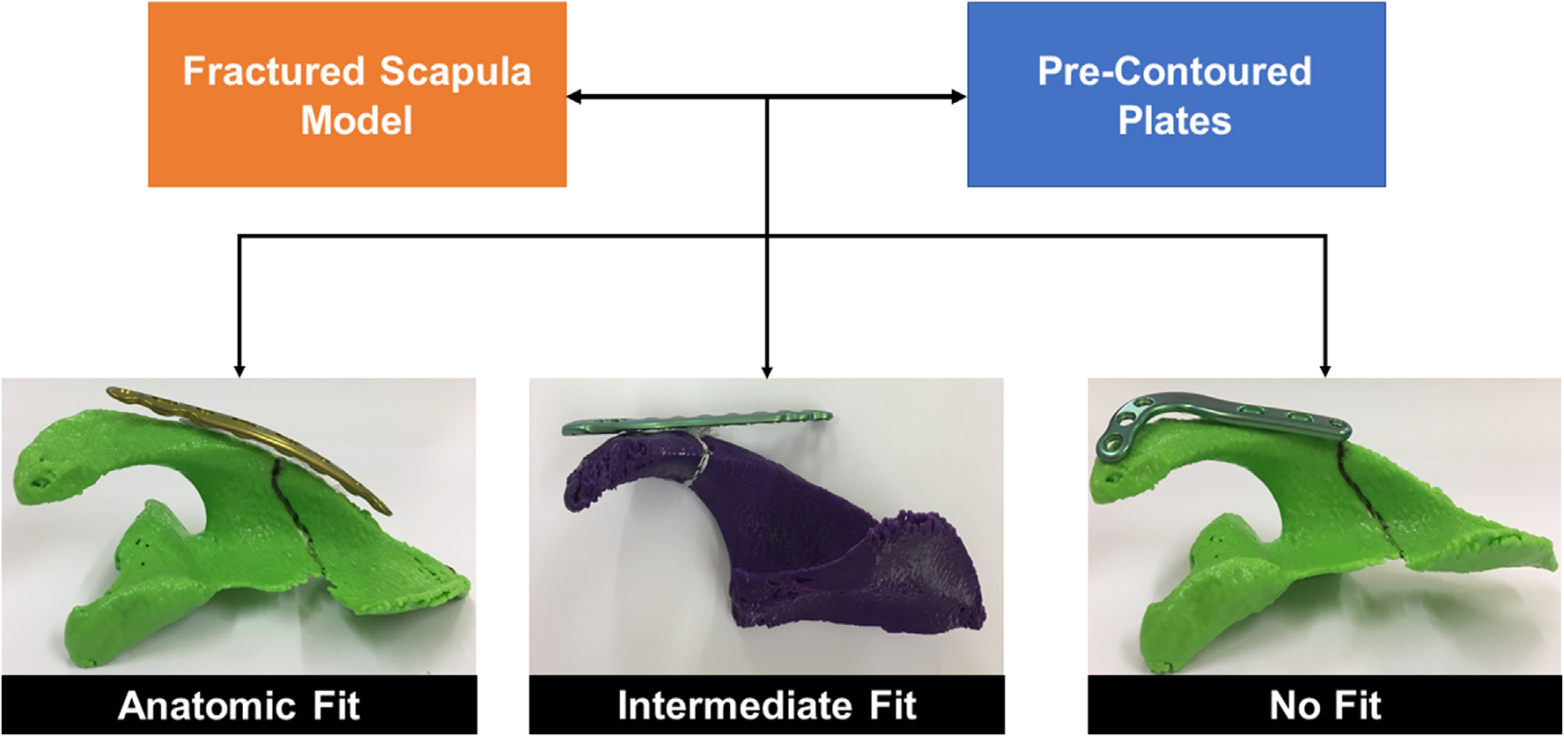
Description of the different classifications employed to analyse the quantitative fit of the scapula plates around a fracture.

A rating approach same as Malhas et al. [16] and previously used in our study on adult museum specimen scapulae [17] was applied where the quantitative fit of the plates was rated according to an ordinal scale (Figure 4). The *anatomic fit* was defined for plates that presented, at least three screw holes on either side of the fracture line and had less than 2 mm bone-to-plate gap; Plates presenting 3 screw holes on either side of the fracture with more than 2 mm bone-to-plate gap with a possibility to achieve anatomic fit by bending, were rated as *intermediate fit*; The plates presenting less than 3 screw holes on either side of a fracture and/or exhibiting significant overhang around the edges of the bone were defined as *no fit.*

Each observation was converted into a numerical score (no fit – 0; intermediate fit – 5; anatomical fit – 10) which quantified the observations. The inter-rater reliability of the ratings provided by multiple observers was determined by calculating the values of the Interclass Correlation Coefficient (ICC – absolute agreement) using the two-way mixed model. The 95% confidence interval (CI) was also calculated for the inter-rater reliability. These statistical analyses were performed using IBM SPSS Statistics v. 25 (Armonk, NY: IBM Corp).

## Results

High ICC values were observed suggesting good to excellent inter-rater reliability. The ICC ranges were observed to be between 0.89 and 0.99 for the first set of observations and between 0.81 and 0.99 for the second set of observations (Table 2). The overall average ICC was found to be 0.965.

**Table 2:** The intra-class correlation coefficient calculated to compare the ratings provided for each scapula during the two rating schedules.

	Ratings during 1st schedule		Ratings during 2nd schedule	
	ICC	CI (lower bound–upper bound)	ICC	CI (lower bound–upper bound)
Scapula 1	0.93	0.808–0.984	0.93	0.813–0.984
Scapula 2	0.91	0.761–0.980	0.98	0.966–0.997
Scapula 3	0.97	0.922–0.993	0.99	0.966–0.999
Scapula 4	0.91	0.740–0.979	0.98	0.948–0.996
Scapula 5	0.96	0.883–0.990	0.98	0.956–0.996
Scapula 6	0.96	0.878–0.993	0.89	0.763–0.981
Scapula 7	0.94	0.835–0.986	0.81	0.744–0.973
Scapula 8	0.96	0.884–0.992	0.93	0.816–0.985
Scapula 9	0.89	0.635–0.968	0.88	0.676–0.973
Scapula 10	0.99	0.987–0.999	0.98	0.932–0.993
Scapula 11	0.99	0.996–0.999	0.97	0.961–0.993
Scapula 12	0.98	0.968–0.993	0.99	0.977–0.998
Scapula 13	0.99	0.982–0.999	0.94	0.877–0.987
Scapula 14	0.99	0.985–0.998	0.99	0.972–0.998

**Figure 5:**
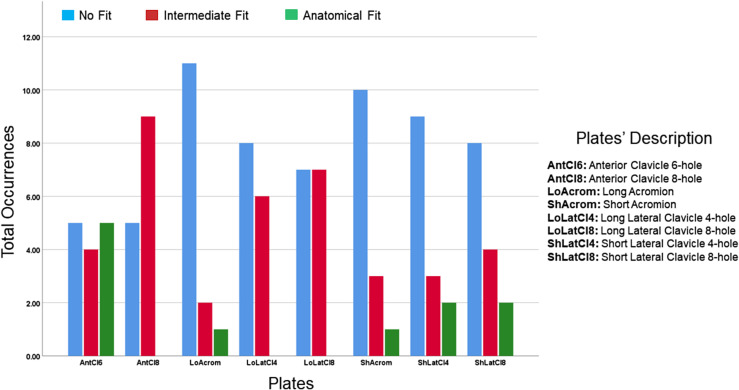
The overall quantitative fit of each plate when templated against acromion and spine fractures.

**Figure 6:**
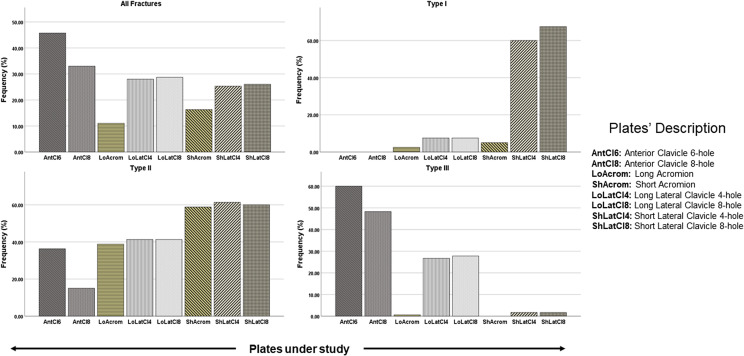
Frequency of the observed anatomical fits for each plate segregated for different types of fractures.

The clavicle plates were found to fit better than the acromion plates. The 6-hole anterior clavicle plate was able to anatomically fit a maximum number of scapulae (*n* = 5). The long acromion plate did not fit most of the scapulae (*n* = 11) followed by the short acromion plate (*n* = 10) (Figure 5). The anterior clavicle 6-hole plate was the plate of choice in 45.7% of cases when all fractures were considered (Figure 6). Lateral clavicle short plates (4- and 8-holes) were the best fit in type I fractures (60.0% and 67.5% respectively) (Figure 6) while the short acromion plate only fitted in 5% of type I fractures. The short acromion plate and the short lateral clavicle (4- and 8-holes) plates showed similar fits (61.3% and 60%) with the best potential to fit type II fractures. The anterior clavicle 6-hole plate had the best potential to fit (60%) followed by the anterior clavicle 8-hole plate (48.3%) in the most common fracture (type III) (Figure 6).

## Discussion

A variety of fixation techniques have been described for the ORIF of acromion fractures, including tension band wiring for more distal fractures, plate fixation for fractures that are more medial or through the acromial base and spine [5, 8], interfragmentary screw fixation [8, 18], plate fixation supplemented with interfragmentary screws [8, 19], and fixation with Kirschner wires [5, 8]. However, published data concerning the decision-making and treatment outcomes of acromion fractures are retrospective and do not compare operative versus non-operative treatment [20]. The latter treatment along with surgical implantation of plates designed for other anatomic regions have been employed at our center to treat acromion fractures before the usage of scapula anatomical plates [21]. There is no evidence, in the literature, regarding pre-contoured [22] scapula plate’s potential to effectively treat the fracture. This problem is more prominent in a developing country like ours where we have limited plating options. In our study, we found that the acromion plates did not provide an adequate number of screw holes on the medial side of the scapula, in the majority of scapular spine fractures. The longer anterior clavicle plate was found to have a better fit around acromion spine fractures, especially the type III fractures. This conforms with our previous observation of long acromion plates having inadequate anatomical fidelity when templated against the scapula spine [17]. Since plate length and screw groove arrangements were the limiting factors for the acromion plates, we would recommend manufacturers modify their plate design to provide more options for fixing medial acromion and spine fractures.

The main limitation of this study is the small sample size. This is due to the low incidence of these fractures. This is however the largest study, in terms of study sample involving acromion fractures, compared to the studies in the literature [1, 11]. The secondary limitation of this study was that it only used plates provided by one manufacturer, as this is the only scapula pre-contoured plating system available in the country. Lastly, the observations from this study were a factor of the morphometry of the scapula included in the dataset. Our dataset included local patients and was male dominant. Observations from previous scapula morphometric studies suggest that gender differences are more prominent for bone size than interpopulation differences [23–26]. The reader should keep these factors in mind while inferring the results.

A wide spectrum of heterogenicity of the acromion anatomy [27–30], and limitations of available implants, together with variable fracture morphology present challenges in trying to achieve favourable fixation and resultant outcomes following surgery. Currently, surgeons incorporate the use of various fixed-angle plate options intended for other periarticular areas in the fixation of scapula fractures. Plate systems for the lateral clavicle have been shown to be a good match for the acromion [1]. The previous study had failed to evaluate the complete clavicular plating system and this was addressed in this study [1]. Painless osteosynthesis of the acromion, post-surgery is very important for the functioning of rotator cuff and deltoid muscles [31]. Pires et al. [13] recommended that all displaced Kuhn et al. [32] classified type II and type III acromion fractures must always be treated surgically to prevent muscle weakness and provide unimpaired functioning of the shoulder joint. In our study, we observed that the clavicle plates had a similar or better fit when it came to medial acromion fractures running into the scapula spine. Therefore, we recommend the usage of clavicle plates for surgical fixations of such fractures to achieve better osteosynthesis and restoration of shoulder activities.

Integrating 3D models into surgical practice might provide an inexpensive avenue to perform pre-operative planning which might also lower the risk of intra- and post-operative complications and improve patient outcomes. Better planning procedures can be expected to improve plate selection for the fracture pattern and therefore optimal fixation. The literature has shown the added benefit of educating patients and empowering them to make informed decisions around accepting a proposed surgical plan when using 3D modelling [14]. Likewise, it can provide similar benefits to surgeons’ education and training [33].

The use of custom 3D printed models of patient bone may become standard in preoperative planning, surgical simulation, intraoperative guidance, and implant development [34]. Results from the work by Bryce et al. further reported that accurate and reproducible 3D models can be created from *in situ* scapulae by use of effective segmentation [35]. This additional evidence reporting on the accuracy of prototype models adds to the global body of work which enables the surgeons to match fracture fragments as done intra-operatively. We recommend the adaptation of 3D modelling and additive manufacturing into pre-operative planning of critical fractures of the acromion and the scapula spine. Anatomically correct restorations of these processes of the scapula are essential for the functioning of important shoulder muscles like the deltoids and the rotator cuff, to prevent diminished shoulder function due to mal-union, and to prevent postoperative complications due to non-union.

The strength of this study is that observers with various levels of orthopedic experience quantified the fit of plates for acromion fractures and achieved good-to-excellent inter-rater reliability. This study was able to highlight the current drawbacks of a pre-contoured plate design and provided evidence that clavicle plates may serve as a better alternative for the acromion process and spine fractures. This is, to our knowledge, the only study that involved the full range of pre-contoured acromion and clavicle plates.

## Conclusion

Currently, pre-contoured acromion plating systems fail to provide adequate congruency and fit for the fixation of most acromion and spine fractures. Our study found that clavicle plates were better at fitting acromion and scapula spine fractures than anatomically pre-contoured acromion plates. The clavicle plates were observed to have a superior chance of fixing medial acromion fractures, ranging from acromion angle into the scapular spine.

## Funding

This research received no specific grant from any funding agency in the public, commercial, or not-for-profit sectors. RD is funded by Harry Crossley Foundation.

## Conflict of interest

The authors had no conflict of interest.
